# The evaluation of disability and its related factors among the elderly population in Kashan, Iran

**DOI:** 10.1186/1471-2458-7-261

**Published:** 2007-09-22

**Authors:** Mohsen Adib-Hajbaghery, Shima Aghahoseini

**Affiliations:** 1 Faculty of Nursing and Midwifery, Kashan University of Medical Sciences, Kashan, Iran

## Abstract

**Background:**

Recent literature indicates that developing countries in Asia are aging faster than other countries in the world and disability has become one of the greater public health concern in these countries. Pausity of published data on the elderly disability in Iran signifies the importance of this study designed to evaluate the disability and its related factors among the elderly population in Kashan, Iran during 2006–2007.

**Methods/Design:**

A cross-sectional study is conducting on a multy-stage random sample of elderly people in Kashan ages 65 years and older. Volunteer participants were included by age 65 and older and excluded if they had the medical diagnosis of Alzhimer disease. The WHO DAS II was used as the generic disability measure in this survey. The original version of WHO DAS II was translated into Farsi according to the standardized guidelines for cross-cultural adaptation of health-related measures. Upon completion of data collection the descriptive statistics will compute all the variables. Chi-square, t-test analysis and ANOVA will be used to examine significant differences between the subgroups.

**Discussion:**

This is the first research protocol to study disability among the Iranian elderly population. Presently, 80% of eligible subjects have been selected. The results of this study will help to develop more effective protocols to assist Iranian elderly population with disabilities.

## Background

The world's population is aging and it is expected the aged population (people over 60s) to reach over two billions mark by the year 2050 [1]. With the countinuing rise in life expectancy, the human life span is now divided into four ages: 1) the age of dependency, childhood, and education; 2) the age of independence, maturity, and responsibility; 3) the period of fulfilment for physical and mental health fit for retirement; and 4) the old age that is associated with disability and dependency [2]. As the population continues to age, the quality of life issues associated with disability become of greater public health concern. The aging of the population and its accompanying burden of disease and disability have profound public health implications for the utilization of medical care, and for the need for health, social and supportive services and long-term care [2,3].

There are several ways of defining disability. The most widely used is the medical definition indicating a disability is "an impairment" or the inability to carry out normal social roles because of the impairment and disability. There is also a social definition that considers the way disabled people's lives are affected by the barriers society imposes on them [4]. It has also defined as an impairment in activities of daily living (ADL) [5], or having a "problem" in performing any of ADLs without help or equipment for three months or longer [6].

Newly developed countries in Asia are aging faster than other countries in the world. The rate of increase of the elderly population age 65 and older in these countries is reported at approximately 3% annually, compared with 1.0% to 1.3% in the United Kingdom (UK), Sweden, and the United States [7]. Based on the report of Iran's Ministery of Health in 2005, the proportion of persons 65 and older in Iran accountes for 6% of the population. It is estimated that this rate will rise to 19% by 2030 [8]. It is also predicted that the population of dependent elderly will reach to more than one third of the total population by the year 2050 [9].

Disabled people use significantly more medical resources than do non-disabled people [10]. Researchers examining the global aging issue in the 1970s concluded that the elderly were increasingly disabled and less healthy [11]. The costs associated with disabilities of the elderly are high and continuing to grow accounting for 3.6% of the UK gross domestic product and some have suggested that overall costs might rise as high as 10.8% by 2030 [12,13].

The rising costs of this problem will negatively effect the quality of life among the elderly people and their physical or mental functioning as well as their families. Therefore, global aging has precipitated policy discussion around world. For instance, Japan has formed a national health plan, Germany has funded a reform for long term care and UK has appointed a Royal Commission to address aging population and their health care needs [2,14].

The study of disability among the elderly by Melzer [2] indicated that 11% of men and 19% of women age 65 and older suffer from some form of disability; 38% were cognitively impaired and more than 80% of disabled elderly needed help at least once a day. Similarly, Minden et al. [15] reported that disability have been costly for elderly by effecting a decrease in their income and an increase in their need for assistance with ADL as nearly 85% of the elderly need help with ADL and 40% with houshold activities.

Researchers have indicated that the prevalence of disability varies in different countries. In a study of social environment and gender differences regarding disability in Egypt and Tunisia, Yount and Agree [16] reported that in Egypt men and women experienced more physical limitations compared to elderly in Tunisia while elderly in Tunisia suffered from multitudes of disabilities including ADL limitations.

Some research literature indicates an increased disability trend in the developing countries [7]. Other published works generally suggest that disability is falling among the elderly in developed and western countries [6,10].

Both chronic diseases and acute events such as cardiovascular conditions, stroke, hip fractures, arhritis, skeletal and mobility problems as well as hospitalization are among the most common underlying reasons for physical disability in older adults [17,18]. Some reports indicate variations in sex and age contributing to the levels of disability [16,19,3]. Parahyba et al. [20] reported that there was a significant relationship between age, sex, low education and low income with the level of disability. Wilcox et al. [18] report that the elderly's perception of disability has more negative effects on their level of health and quality of life than their actual level of disability.

All these findings support the significance of this study and show the necessity for an effective protocol addressing disability health issues among the elderly in Iran. Studies of the prevalence, causes, and effects of disability in the aging population are crucial for an appropriate public health policy and planning in Iran. Whereas many population studies on disability in older adults have been conducted in western countries, this study will enhance the health care providers' understanding of elderly disability issues in Iran and provide a closer reflection on similar cultures in Asia and the Middleastern countries.

While the Iranian population of elderly people is rapidly increasing and the population is shifting age pattern from young to adult [8], the government and health care system have not focused on the demandes of elderly as a voulnerable subgroup of the society. A large number of aging adults in Iran are illiterate, 70% live in cities [8] and need more attention by the national health care system. The health condition of Iranian elderly and their types and levels of disabilities have not been studied before. This study was designed to evaluate the disability and its effects among the elderly population in Kashan, Iran during 2006–2007.

## Methods/Design

A cross-sectional study is conducting on a random sample of elderly people (ages 65 and older) residing in Kashan, Iran.

### Setting, sample and inclusion criteria

Kashan is a large city in Isfahan province (at the center of Iran) and located 200 KM from Tehran (the capital of Iran). Based on the last census data in the year 2000 it has been estimated that approximatly 25,000 people living in Kashan, Iran are 65 years and older. There are 35 health centers within the city addressing the primary health care issues of the entire city under national health care plan.

Sample size was calculated based on the previous report by Melzer [2] who estimated 30% of aging population have some form of disability. The formula listed below was used [p = 0.30, q = 0.70, d = 0.05, a = 0.95] for 350 subjects in the study [n=z2∗pqd2]
 MathType@MTEF@5@5@+=feaafiart1ev1aaatCvAUfKttLearuWrP9MDH5MBPbIqV92AaeXatLxBI9gBaebbnrfifHhDYfgasaacH8akY=wiFfYdH8Gipec8Eeeu0xXdbba9frFj0=OqFfea0dXdd9vqai=hGuQ8kuc9pgc9s8qqaq=dirpe0xb9q8qiLsFr0=vr0=vr0dc8meaabaqaciaacaGaaeqabaqabeGadaaakeaacqGGBbWwcqWGUbGBcqGH9aqpdaWcaaqaaiabdQha6naaCaaaleqabaGaeGOmaidaaOGaey4fIOIaemiCaaNaemyCaehabaGaemizaq2aaWbaaSqabeaacqaIYaGmaaaaaOGaeiyxa0faaa@3A8A@.

Having 65 years of age or older, willing to participate in the study and not having a medical diagnosis of Alzhimer disease helped with a multy-stage random selection and sampling strategy. The reason for excluding subjects with the diagnosis of Alzhimer was based on the necessity to have clear cognition of all participants.

**Figure 1:**
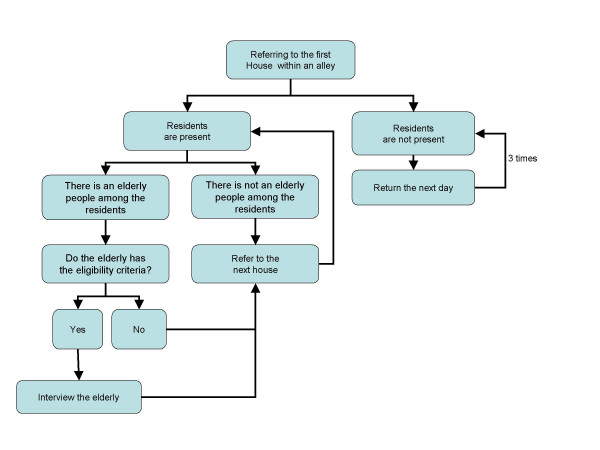
Process of sampling in each cluster.

The demographic map of the city was divided into 35 region based on the population covered by each health care facility. Each region had between 1–3 section. One or two alleys selected in each section and subjects were selected from residenence of homes in each alley. Subjects were selected by age 65 and older and assesed for exclusion criteria [fig. 1]. The researcher identified potential subjects, introduced study purpose and obtained voluntary informed and written consent. Subjects completed a questionnaire and engaged in a formal interview. At this time 80% of data has been collected and being entered for computation. The sampling will continue untill the required sample is reached.

### Questionnaire

The WHO DAS II 36 items was used as the generic disability measure in this survey. The WHODAS II has been developed to assess the activity limitations and participation restrictions experienced by an individual irrespective of medical diagnosis. The WHO DAS II explores what people do in different areas of life using 36 items that ask for respondents to state the level of difficulties experienced in six domains of life during the last 30 days. The domains included in the instrument are: Understanding and communicating (cognition), Getting around (mobility), Self care (attending to one's hygiene, dressing, eating and staying alone), Getting along with people (interpersonal interactions), Life activities (domestic responsibilities, leisure, and work) and Participation in society (joining in community activities). Self-reported health status was also assessed using a single question ("In general, would you say your health is excellent, very good, good, fair or poor?").

Socio-demographic data included age, sex, living status, needing help in activities, marital status, insurance status, education, employment status, addictions (dependency), current and previous work status, household income, housing type (a reliable surrogate indicator of socioeconomic and income status), living arrangement, having regular bodily activities, and caregiver availability.

### Medical Conditions

Subjects were asked to report their most recent state of wellbeing within the last 12 months before the interview and list of any specific medical conditions, which included coronary artery disease, heart failure, hypertension, dyslipidemia, diabetes mellitus, stroke, cancer, hip fracture, arthritis, asthma, chronic obstructive pulmonary disease (COPD), cataract, and other conditions. The frequency of hospitalization [for every medical condition reported by the respondent] in the 12-month period before the interview was also collected.

### Translation of WHO DAS II

The original version of WHO DAS II was translated into Farsi according to the standardized guidelines proposed by Guillemin et al. [21]. A native English speaker living in Iran who understood Farsi language quite well and did not have indepth knowledge of disability assessment carried out back translation. The final version derived from reconciliation of the original and back translation and tested on 15 elderly people. The content validity of translated WHO DAS II was approved by 10 faculty members in Kashan Medical University. Translated WHO DAS II also was repeated in 15 before mentioned people in 2 weeks apart for test-retest analysis. Reliability was determined from Cronbach's alpha and test-retest. Cronbach's alpha was higher than 0.91 for domains and it was 0.93 for overall scores. Spearman's rank correlation was also 0.89 for WHO DAS II and it was higher than 0.73 for WHO DAS II domains.

### Statistical analysis

The WHO DAS II has a likert scale format rated on a scale of 1–5 [from none to extreme disability], however, the researchers will rate it on a scale of 0–4 [0 = none, 1 = Mild, 2 = Moderate, 3 = Severe and 4 = Extreme disability or cannot do]. The maximum score is 144 and the minimum score 0. The participants' total score will categorize under the 5 categories of extreme, sever, moderate, mild and without disability for the score levels of 0–36, 37–72, 73–108, 109–143 and 144 respectively. Descriptive statistics will computed for all variables. Chi-square, t-test analysis and ANOVA will also utilize to check significant differences between the subgroups.

This study was granted Institutional Review Board (IRB) approval by Kashan University of Medical Sciences [KAUMS] and received ethics approval from the ethic committee of KAUMS. All subjects were provided a copy of the written consent and assured of their annonymity and confidentiality of data obtained. Current raw data has been secured in a locked cabinet at researcher's office.

## Discussion

Disability due to physical, mental, or emotional health problems is a major public health issue, resulting in the reduction of life quality and increased dependence on the health-care system. Disabilities are disproportionately represented among the elderly population and those with lower socioeconomic status [3]. The capacity of health system to be prepared for a public health threat is influenced not only by the community in which the health care system is located but also by the data the system has about the community. This study will provide the first data set on this subject.

To date, this is the first research protocol to study disability among the Iranian elderly population. This population is particularly at risk not only for changes in their bodily mechanisms but also for the lack of authority attention to the rapid increase of aging population and their health care needs. Although different disability measures exist, for this study researchers selected the WHO DAS II questionnaires for its cultural appropriateness to the Iranian population. The ease of application to a larger sample size also played a role in selection of this instrument. The pending results of the data analysis will help the health care providers gain more insight into the scope of disability among the Iranian elderly.

### Progress of the study

Presently 80% of eligible subjects have selected for inclusion and data collection. Available data is entered into the statistical software for later analysis. The results of this study will be available in 2008.

## Competing interests

The author(s) declare that they have no competing interests.

## Authors' contributions

MAH contributed to the development of the study protocol, data collection, analysis and preparing the report and manuscript. SAH contributed in data collection and entry to the statistical software. Both authors have read and approved the final manuscript.

## Pre-publication history

The pre-publication history for this paper can be accessed here:


